# The Role and Welfare of Cart Donkeys Used in Waste Management in Karachi, Pakistan

**DOI:** 10.3390/ani9040159

**Published:** 2019-04-12

**Authors:** Syed Zahir Ali Shah, Zahid Nawaz, Sher Nawaz, Gemma Carder, Manuchahar Ali, Naimatullah Soomro, Polly C. Compston

**Affiliations:** 1 Brooke (Pakistan) South Region, RCVH, MA Jinnah Road, Karachi 74100, Pakistan; zahid@thebrooke.org.pk (Z.N.); shernawaz@thebrooke.org.pk (S.N.); manuchahar@gmail.com (M.A.); naimatullah.soomro@thebrooke.org.pk (N.S.); 2 Brooke (UK), Friars Bridge Court, 41-45 Blackfriars Rd, South Bank, London SE1 8NZ, UK; gemma.carder@thebrooke.org; 3 The Royal Veterinary College, Hawkshead Lane, Hatfield, Hertfordshire AL9 7TA, UK; pcompston@rvc.ac.uk

**Keywords:** cart donkey, animal welfare, waste management, Karachi, Pakistan, working equid

## Abstract

**Simple Summary:**

In low and middle-income countries, equids (donkeys, horses, and mules) play a critical role in supporting people’s livelihoods. In Karachi, donkey carts are used to transport building materials, commercial produce and garbage. However, to date the role of donkeys in waste management has received little attention. This study therefore aimed to articulate the role and welfare of donkeys used in waste management. Interviews were conducted with donkey owners, households that use donkey carts to collect their waste and key informants. We found that the collection of waste was the primary source of income for 89% of donkey owners interviewed; of those working in waste management, 62% were under 18 years of age. Households reported removal of waste by donkey cart as their preferred waste management option, and reported that there would be a huge garbage build-up if donkey carts became unavailable. A number of animal welfare concerns were documented; 52.9% of donkeys had a body condition score of two. Muzzle mutilation was extremely high (78.4%) and 66.7% of donkeys had superficial knee lesions. We recommend that appropriate public resources are allocated to improve the welfare of both donkeys and people working in waste management in Pakistan.

**Abstract:**

Equine ownership is a common income-generating strategy in Pakistan. In Karachi, donkey carts are used to transport building materials, commercial produce and garbage. This study aimed to articulate the role and welfare of donkeys used in waste management. We conducted interviews with donkey owners (*n* = 200), households which use donkey carts for waste collection (*n* = 50) and key informants (*n* = 14). To assess the welfare of donkeys, the Standardised Equine-Based Welfare Assessment Tool (SEBWAT) was used (*n* = 204). Collection of waste was the primary source of income for 89% of owners interviewed. Of those directly involved in waste collection, 62% were found to be under 18 years of age. During interviews with donkey cart customers the majority reported that there would be a huge garbage build-up if donkey carts were not available. Welfare assessments demonstrated that 52.9% of donkeys had a body condition score of two. Muzzle mutilation was extremely high (78.4%) and 66.7% of donkeys had superficial knee lesions. This is the first study that has explored the role of donkey carts in waste management in Pakistan. The data demonstrate the sizable role that donkey-owning communities play in waste management and the important livelihood option this offers, as well as considerable animal welfare concerns.

## 1. Introduction

### 1.1. Waste Management

The management of solid waste (process of gathering, collecting, transporting, segregating and disposing of waste material); [1] is a recognized problem in cities throughout the world [2,3,4]. An increasing global population, combined with rapid urbanization and industrialization have changed the quantity and composition of solid waste generated [3]. As a result, waste management systems in low- and middle-income countries (LMICs) are often inefficient, due to low collection coverage, irregular collection services and open dumping [5]. Current methods of municipal solid waste management cause a variety of economic, public health and environmental concerns [3,6]. A typical example has been described for La Paz, Bolivia, where an informal sector of vulnerable and marginalized waste pickers are primarily responsible for recycling in the city, yet their contribution is unrecognized by the public sector and they often work in poor conditions and for little pay [6]. Additionally, as a result of having direct contact with waste and a lack of protective clothing and equipment, waste pickers are prone to health risks [7]. Landfills are often the only method of waste disposal [3]. However, as is the case in Malaysia, when landfills are poorly designed they pose serious environmental and public health threats [3] and are a major contributor to greenhouse gas emissions [8]. Further concerns include antimicrobial resistance; one study found that in Norway red foxes living in urban areas were more prone to exposure to resistant bacteria and resistance drivers from garbage and sewage as compared to foxes living in rural areas [9].

### 1.2. Waste Management in Pakistan

In Pakistan the management of municipal solid waste is often inefficient; approximately only 60% of solid waste is collected, resulting in uncollected rubbish gathering in the streets [2,4]. Karachi is Pakistan’s largest city, with a population of over 20 million and more than 14,000 tonnes of solid waste produced daily [10]. As with all major cities in Pakistan, the current methods for solid waste management in the city are inadequate, resulting in significant difficulties for its citizens [2,10,11]. These problems have multifaceted causes, generally associated with ineffective management of waste including shortage of trained manpower, lack of reliable data and poor administration [12]. In Pakistan, solid waste collection is primarily governed by municipalities [11], and waste is collected door to door for a fee of between $0.71 and $19 United States Dollar (USD) per household per month by private ‘waste pickers’ [10]. Recyclable materials such as metal and plastic are segregated by home-owners and/or waste pickers and sold for revenue [10]. Non-recyclable waste is typically transported to large containers designated by the town municipal administration, and subsequently moved to landfills. Unauthorized dumping at other sites is reported to occur throughout Karachi [10].

### 1.3. The Role of Equids

Globally there is an equine population of approximately 116 million [13], and approximately 112 million are working equids [14], used in LMICs to support people’s livelihoods [15]. Equine ownership is a common income-generating strategy in Pakistan, which has the third largest donkey population in the world and a total of 5.8 million working equids [13]. These animals generate a household income of $3000 USD per month in some areas of the country, and throughout are important sources of traction and transport, especially within communities with lower socioeconomic status [15]. Throughout Karachi, donkey carts are abundant; they are used in the transportation of building materials and fresh produce as well as for garbage removal. 

Waste collectors use working equids in many LMICs [16]. Previous work by equine welfare organizations has often been difficult because of these communities’ marginalization, which results in a lack of trust in outside assistance and difficulty accessing recognition and resources from public sources [17,18]. To date, the role of cart donkeys and their owners in Karachi’s waste management system has received little attention. This study aimed to assess the role and welfare of cart donkeys used in waste management in Karachi, and understand the challenges that communities face, to help aid the development of appropriate and applicable interventions at a community and citywide level.

## 2. Methodology

### 2.1. Ethics

Ethical approval was gained from Brooke’s Animal Welfare Ethical Review Body. Written and/or verbal informed consent was gained from all participants.

### 2.2. Study Location

The data for this study were collected between 20 July and 25 November 2016. Interviews and donkey welfare assessments were conducted in the Jamali Goth neighbourhood in Karachi, Pakistan. All interviews and questionnaires were conducted in Urdu or Pashto.

### 2.3. Interviews

#### 2.3.1. Donkey Owner Questionnaires

Donkey owners were interviewed by three researchers using a pre-designed questionnaire (Supplementary Material 1). Inclusion criteria included cart donkey owners who used their donkeys for waste management. Exclusion criteria included owners who: were not willing to unhitch their donkey during the questionnaire, had another household member participating in the study or were under 18 years old. There were three main exits to the Jamali Goth community, which were used as convenient places to interview donkey cart owners. Data collection took place when the majority of the donkey owners returned from work (11:00–16:00). Every tenth donkey owner was questioned, with the first selected randomly. Prior to administering the questionnaire, the interviewer(s) ensured that the interviewee’s donkey was unhitched from the cart, offered green fodder and had access to shade and clean water. The questionnaire gathered data on donkey health and welfare and the participants’ role in collecting waste.

#### 2.3.2. Household Questionnaires

Questionnaires were administered to 50 properties in Karachi that have waste collected by donkey carts (Supplementary Material 2). The properties were randomly selected within seven districts in Karachi; first by randomly generating 50 coordinates, followed by the selection of each property with a spinning pencil. Properties were only included if the respondents were a resident or working within the property and had a contract with the town council to collect the property’s waste by donkey cart. One adult member from each household was interviewed. The questionnaire covered preferred waste disposal method, way of obtaining waste disposal services and available alternatives.

#### 2.3.3. Key Informant Interviews

Key informant interviews were conducted with eight town committee workers and six local animal healthcare practitioners.

Members or associates of town committees in two main areas (New Karachi and North Nazimabad) were interviewed. These locations were purposively selected based on their size (one large and one smaller council). Three sanitary inspectors, two directors, one union council chairman and one contractor were interviewed from the two areas. Briefly, the interview elicited information about the municipality’s waste management process, which agencies were responsible for the different aspects of waste management and the advantages and disadvantages of donkey-driven waste management (Supplementary Material 3).

Animal healthcare practitioners providing services to the donkeys in Jamali Goth were identified following discussion with community members and interviewed. Briefly, the interview covered common clinical presentations seen and the animal healthcare practitioners’ relationship with donkey owners (Supplementary Material 4).

All key informant interviews were conducted in Urdu, recorded and simultaneously translated and transcribed into English.

### 2.4. Donkey Welfare Assessments

Welfare assessments were performed using the Standardised Equine-Based Welfare Assessment Tool (SEBWAT), within which 40 animal-based indicators are collected [19]. Data collection occurred between 20 and 25 November 2016. Data collection was conducted by two Brooke-trained welfare assessors working together. In line with the recommended methodology for the tool, one assessor examined each animal, while the other verified the scores [19]. The assessors regularly switched roles to reduce the risk of error through fatigue [19]. Data were collected on paper and transferred to a database for analysis. Cart donkeys were randomly selected at three main exits from the Jamali Goth community. Refer to Table 1 and Table 2 for definition of body conditions score and scoring criteria for lesions.

### 2.5. Data Analyses

Quantitative data were analysed using pivot tables and descriptive statistics in Excel. Qualitative data were analysed thematically.

## 3. Results

### 3.1. Questionnaires

The most relevant data collected from the questionnaires are presented.

#### 3.1.1. Donkey Owner Questionnaires

**Figure 1:**
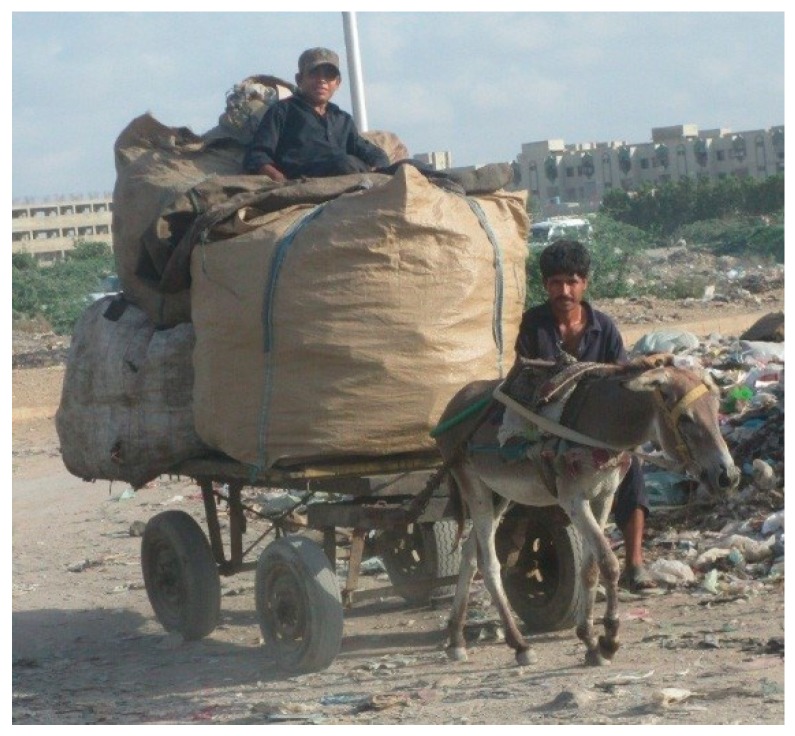
A typical donkey cart waste management team.

Questionnaires were administered to 200 donkey owners. Each participant owned 1–3 donkeys (1 = 78%, 2 = 22.5% and 3 = 3.5%). All owners used their donkeys to collect waste. Collection of waste was the primary source of income for 89% of respondents (Figure 1). The median monthly income received by donkey cart owners for waste collection was 7000 Rs ($50; range 0–68,000 Rs) per month.

On a daily basis each cart donkey (*n* = 255) transported on average (median) 1000 kg of non-recyclable waste and (median) 100 kg recyclable waste (Table 3). The median amount of recyclable waste collected on a monthly basis was 3142 kg (range 0–36,000). One-hundred-and-thirty-seven interviewees (68.5%) reported dumping waste at official sites; 48 (24%) at unofficial sites and 15 (7.5%) declined answering this question.

The median number of members in each household was ten (range 1–41). Several household members would be involved in waste management: the household head would be accompanied by two others (range 1–6 people in total) when collecting waste, and three (median; range 0–8) would be involved in sorting recyclable waste back at the family’s settlement. Of those directly involved in collecting waste each day, 62% were under 18 years of age and 47% were under 14 years of age.

**Figure 2:**
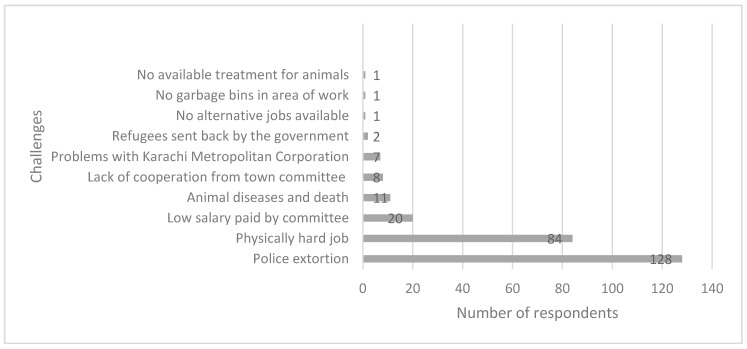
Challenges reported by donkey cart owners during waste management work. Note some participants gave more than one response, 283 challenges were reported.

Household heads reported that police extortion was the most frequent challenge encountered during their work (Figure 2).

**Figure 3:**
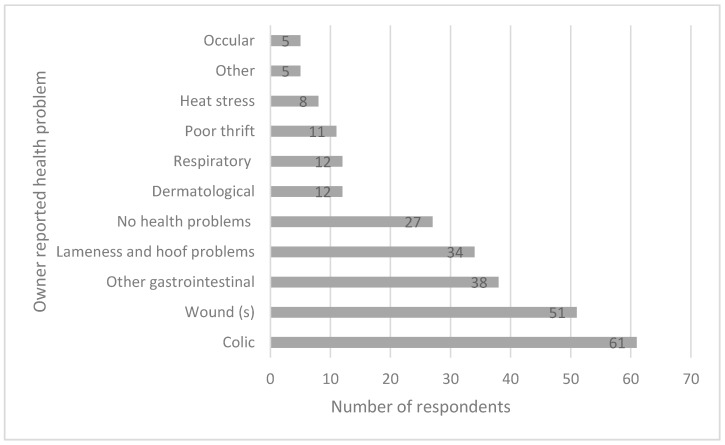
Owner-reported health problems in donkeys over two years prior to questioning. Note that some participants gave more than one response.

The majority of respondents (82.5%) indicated that if their donkey(s) died they would purchase another one to use for waste collection. Donkey owners were asked what the last health problem(s) was with their donkeys (Figure 3). The majority (63%) reported a single problem with their donkeys, 25% reported two problems and 2.5% reported three problems, while 10% of owners reported no problem. Colic was the most frequent health problem reported (Figure 3).

#### 3.1.2. Household Questionnaires

**Figure 4:**
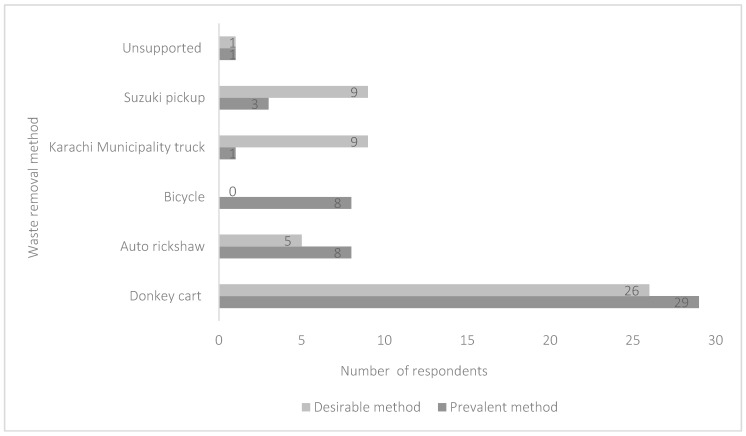
Reported prevalent and desirable waste management systems reported by questionnaire respondents who employ donkey cart owners to remove waste from their property.

Questionnaires were administered to 50 participants living or working in properties who had a contract with the town council to collect the property’s waste by donkey cart. All were residential properties except for one, which was a shop. All respondents except for one said that waste was collected daily by donkey cart owners, and the majority (52%) stated that a donkey cart was their preferred methods of waste collection (Figure 4). When asked what would happen if a donkey cart did not visit their residence, 53% said that there would be a huge garbage build up, 25% said there would be unpleasant smells and 23% reported that the conditions would be unhygienic within one month of waste not being collected. If donkey cart owners stopped working in the respondents’ area 56% said that they would employ another donkey cart owner to collect household waste, 28% said they would dispose of their waste themselves and 16% would contact the town committee for an alternative option.

### 3.2. Key Informant Interviews

#### 3.2.1. Town Committee Workers

Three sanitary inspectors, two directors, one union council chairperson and one contractor were interviewed. They described their role as contracting waste collection services for residential and commercial properties in their district, as well as ensuring that municipal dumps were used and managed appropriately. They managed household-level waste collection services through contracting private individuals and therefore had very little, if any, contact with the donkey owners. Interviewees reported a perception that these private individuals would try to maximize their profit by employing the cheapest labour available to perform waste collection. There was no consensus on the agency that ultimately governed waste management in the city; additionally, most were not aware of a waste management policy for Karachi. Challenges discussed associated with waste management included lack of resources for equipment and manpower, the inappropriate sewage system in place and the narrow streets of the city. When questioned about the future of the donkey cart in Karachi, several thought that they were likely to be replaced with motorcycles, or that some alternative solution would be required to ensure that waste management was sufficient for the growing population.

#### 3.2.2. Animal Healthcare Practitioners

Five veterinarians and one para-veterinarian were interviewed, all of whom provided services to the community of donkeys at Jamali Goth, among other species and locations. Commonly reported clinical presentations included urine retention, colic, lameness and gastrointestinal impaction due to plastic bag ingestion. In particular, cases of diarrhoea, lameness and colic were thought to be more common in this population of donkeys compared to other donkeys in Karachi. Those interviewed encountered some difficulty in providing healthcare to the donkeys in the Jamali Goth community: owners did not have confidence in their treatment, and would request compensation if treatment was unsuccessful. Donkey owners often were too far away from the animal healthcare practitioner, or had insufficient funds to pay for treatment. This difficult relationship was compounded by the language barrier experienced as the donkey cart owners were often ethnic Pathan from Afghanistan. 

### 3.3. Donkey Welfare Assessments

The health and welfare of 204 donkeys were assessed (stallions *n* = 201, mares *n* = 3). Data collected on body condition score and lesions are reported, as in this study these two SEBWAT indicators demonstrate the most severe welfare compromise.

**Figure 5:**
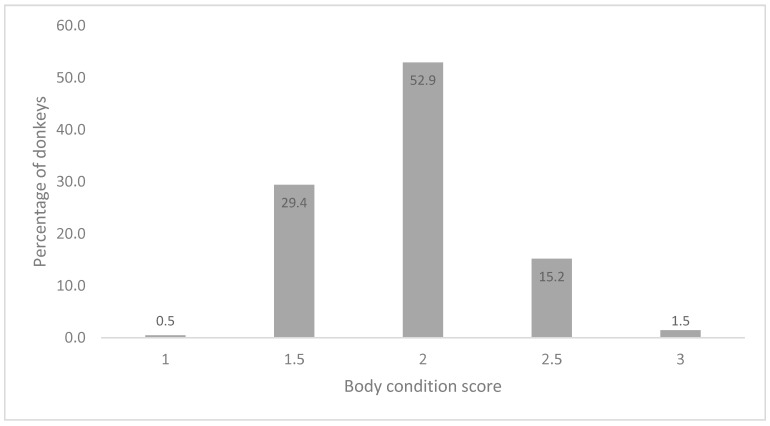
Body condition score for donkeys included in the assessment, scores of 4 (fat) and 5 (very fat) were not given.

Using SEBWAT, each donkey’s body condition was scored (Figure 5). Refer to Table 1 for scoring criteria.

The donkeys were assessed for mutilations; occurrence of muzzle mutilation was high (78.4%). A small percentage of donkeys had experienced tail mutilation (0.98%) and ear mutilation (15.7%). Superficial and open lesions were also recorded (Table 4). See Table 2 for scoring criteria. Twenty-two (10.8%) donkeys had no wounds, and the rest had one or more wounds.

## 4. Discussion

### 4.1. The Role of Cart Donkeys in Waste Management

The study aimed to articulate the role of cart donkeys and their owners in Karachi’s waste management system and assess the welfare of the donkeys working within it. The results demonstrate the sizable role that these animal-owning communities play in waste management and the important livelihood opportunity it offers. Donkey cart owners included in this study were asked how much recyclable and non-recyclable waste they collect on a daily basis. Each day on average each cart donkey transported 1000 kg of non-recyclable waste and 100 kg of recyclable waste. Different families occupied different niches, with some collecting large amounts of one particular type of recyclable material (see Table 3 for the large ranges for each category recorded). Donkey carts are the most prevalent and desirable method of waste removal in Karachi. Although the number of donkey carts engaged in rubbish collection in Jamali Goth is unknown, this community is likely to be responsible for the removal of huge quantities of waste in Karachi. However, at least 24.5% of this waste is being dumped at unofficial landfill sites. It is likely that this is to mitigate the effects of having to carry heavy loads over long distances. Mapping the location of where donkey cart owners are living, which houses they are providing waste for and the location of landfill sites could help to plan waste management journeys made by cart donkeys efficiently, reducing the distance that the donkeys and their owners had to travel every day and providing an incentive to use official sites for dumping waste.

The important role that donkey cart owners play in waste management in Karachi was further demonstrated when questionnaires were administered to 50 participants living or working in properties who had a contract with the town council to collect the property’s waste by donkey cart. All respondents indicated that there would be a huge rubbish build up and/or unhygienic conditions/bad smells if waste was not collected. Waste removal by donkey cart was reported to be the preferred method of waste removal by the majority of the respondents (56%). This could be because the narrow streets of Karachi preclude most other options currently available for curbside waste collection. Any motorized alternatives (for example auto-rickshaws) are unable to carry the amount or weight possible with a donkey cart. Despite the welfare compromise that can occur, this means that it is difficult for either the cart owners or the municipality to use alternative means of transport.

Eighty-nine percent of donkey cart owners stated that collection of waste was their primary income source. This income supports large families (median 10 members), many of whom were directly involved in collecting waste each day. Unfortunately, 53% of those actively involved in the collection of waste were under 18 years of age, with 47% being under 14 years of age. Waste collection in LMICs often involves children and working in this industry can prevent them from having a formal education [20]. Child labour within the waste management system adds a layer of complexity to any intervention that seeks to support their work. There would be benefits to collaborative intervention with agencies that work to build access to education for these children.

The legal age for workers in Pakistan is 18 years, and therefore any policy ask would need to be within the bounds of Pakistan law. Although waste management is ultimately a responsibility of the town’s District Municipal Corporations, they contract this to intermediaries who are responsible for providing waste collection to households within their district. Ultimately, this creates a lack of transparency and amplifies the marginalization of this group of immigrants doing a low-status job.

### 4.2. Animal Welfare

Using SEBWAT the welfare of donkeys was assessed. The body condition of 204 donkeys was scored; the most common body condition score (BCS) was 2 (52.9%) followed by 1.5 (29.4%). A score of 2 indicates that the donkeys were considered ‘thin’ demonstrated by a concave neck, with prominent ribs, hooks, pins and tail-heads. A score of 1.5 is intermediate between ‘very thin’ and ‘thin’ (see Table 1). Studies that have assessed the BCS of working equids in LMICs have found similar results to this study. For example, in one study the BCS of equids used for draught, pack and ridden work in Afghanistan, Egypt, India, Jordan and Pakistan was assessed, and data showed that 70% of equids were thin, having a BCS of 2 or less [21]. These animals are likely to be thin because of their extremely high workloads (both in terms of the long distances travelled and large weights carried daily) and poor nutrition [22]. Subclinical infectious disease may also be a contributing factor. When donkey owners were asked which health problems their donkeys had experienced, gastrointestinal problems were reported by owners as the most prevalent health issue (55.5%), followed by work-related injuries (28%). This was validated by the animal healthcare practitioners who reported a high rate of colic and impaction (often attributed to plastic bag ingestion), indicating that these animals are not receiving adequate diets. Additionally, animal health practitioners reported high rates of urine retention. This is a common misdiagnosis for gastrointestinal pain in working equids, illustrating there are likely to be capacity gaps in the animal health practitioners treating these animals, and also that the rate of colic and gastrointestinal disease may be higher than reported [22].

Occurrence of muzzle mutilation was high (78.4%). Mutilations are often performed in working equids for cultural, identification, husbandry or perceived therapeutic reasons [19,23]. Mutilations are a common welfare problem in working equids in many countries; for example one study conducted in India found that mutilations comprising nose-splitting, ear splitting and branding were the most common type of skin wound experienced by equids (comprising 62.8% of all wounds identified) [24]. Nostril slitting has been reported previously in Karachi [25,26,27]; this occurs due to the belief that the airways of animals in respiratory distress can be widened through the practice of creating a vertical slit from the ventral aspect of the nares up into the nostril, thereby alleviating dyspnoea during heavy work. Successful community interventions have been reported to mitigate this practice through influencing key community leaders and building community understanding of the natural anatomy of the respiratory tract [22,25]. Lesions were recorded, with a high percentage of donkeys (66.67%) suffering from superficial knee lesions. In LMICs lesions are a commonly reported welfare issue in working equids [19,24,28]. The high prevalence of knee lesions found in this study in particular may be due to overloading, marshy and slippery streets and large quantities of garbage in the streets. Interestingly, wounds were reported as a concern by donkey owners but not by the animal health practitioners, which may indicate a gap in appropriate wound management.

### 4.3. Limitations and Future Research

To our knowledge this study represents one of the first socioeconomic analyses that focuses on working animals within a municipal waste management system. However, some limitations should be taken into account. Some financial and transaction data were difficult to obtain because of the sensitivity surrounding their collection. As discussed, the communities that own these donkeys are marginalised and it is important not to elicit information from them that will compromise their livelihoods further. Two-hundred donkey owners were interviewed at the three main exits from the Jamali Goth community. In order to gain a deeper understanding of the role of donkey cart owners in waste management, future research could replicate this study in other areas within Karachi and other major cities in Pakistan. Furthermore, 50 questionnaires were administered to properties that have waste collected by donkey carts; this data has provided an insight into household head’s preferred method of waste collection in Karachi. In the future it would be beneficial to administer further questionnaires to households in other cities in Pakistan. This study was conducted in a low-resource context, there may have been an inherent imbalance of power between the researcher and the participants, which may have resulted in a certain level of response bias.

The welfare assessment data highlighted concerns about the welfare of the donkeys used for waste management. The causes of the identified welfare problems are likely to be multifactorial. Involving this community in identifying potential solutions to these problems would be a first step towards creating sustained welfare improvements. In addition to the health risks to animals, it is well documented that in LMICs there are human health and safety risks associated with working in waste management [20]. Occupational health risks to waste pickers in LMICs are often high due to manual handling of waste and a lack of protective clothing, such as gloves [20]. Due to lack of resources the health and safety of the donkey cart owners included in this study were not assessed; in the future it would be useful to quantify common health concerns and injuries that donkey cart owners experience. Such understanding would aid the development of preventative measures.

### 4.4. Recommendations

The results from this study show that the welfare of the donkeys involved in the waste management system of Karachi is intimately associated with the livelihoods of their owners. Any intervention that aims to improve welfare must consider this. We recommend a multi-agency response that will address the social issues that occur in this community alongside animal welfare. It is clear that donkey cart owners contribute significantly to Karachi’s waste management, and should be considered when governing organisations are considering both waste management policy and also service provision for these workers and their animals. We recommend that a necessary first step is for the authority to recognise the critical role that donkey cart owners play in the waste management system in Karachi. In relation to the role of informal sector recycling in waste management, it has been suggested that policy makers are increasingly recognising the positive role of the informal sector [20]. It is suggested that planning of municipal solid waste management needs to place more importance on understanding and building on existing informal collection and recycling systems [20]. We recommend that government and non-government organisations work to build an enabling environment within which marginalised communities can act to represent themselves and claim access to their right to health care and education. This should include access to appropriate and competent animal healthcare services.

## 5. Conclusions

This is the first study we are aware of that has explored the role of donkeys and their owners in waste management in Pakistan. The findings highlight the very vital and key role of cart donkeys in waste management and the livelihood opportunity this activity presents to donkey owners. Cart donkeys are a convenient and cost-efficient system of waste management compared to other waste management systems, and represent the primary source of income for the majority of donkey cart owners. The study also highlights the animal welfare concerns present within this population, such as low body condition score, mutilations, wounds and inadequate animal healthcare provision. Results from this study certainly warrant further investigation in other major cities in Pakistan. We recommend that relevant stakeholders work together to ensure the role of cart donkeys and their owners are recognized in public planning exercises, and that appropriate public resources are allocated to improve the health and welfare of both donkeys and people working in waste management in Pakistan.

## Figures and Tables

**Table 1:** Scoring criteria for body condition. If all criteria for a full score were not attained, a half score was awarded [19].

Body Condition Score	Description	Scoring Criteria
1	Very thin	Neck concave; pelvis hollow; shoulder point, spine, ribs, hooks, pins and tail-head are prominent.
2	Thin	Neck concave or straight; pelvis flat; shoulder point, spine, ribs, hooks, pins and tail-head are visible.
3	Medium	Neck straight; point of shoulder not clearly visible and joins the body smoothly; spine slightly visible at withers but smooth elsewhere; ribs not visible; pelvis well filled and slightly rounded; tail-head slightly visible, but well filled and joins the rump smoothly.
4	Fat	Neck slightly convex; some fat accumulation behind shoulder; slight ‘gutter’ along spine; some fat accumulation over ribs; pelvis well rounded or slightly ‘heart-shaped’; some fat accumulation over the tail-head.
5	Very fat	Neck distinctly convex; fat accumulation behind shoulder clearly visible; fat accumulation on either side of spine with a distinct ‘gutter’; fat accumulation clearly visible over ribs; pelvis distinctly rounded (clearly ‘heart-shaped’); fat accumulation clearly visible over the tail-head.

**Table 2:** Scoring criteria for lesions [19].

Lesion Score	Description	Scoring Criteria
0	None	No lesion in the specified body area, or there are only severity Score 1 lesions of less than the minimum qualifying size of 4 cm^2^.
1	Superficial or healed lesion	Superficial or healed lesion, including hairless skin, which may be pale pink if partially broken, scabs or scar tissue, greater than 4 cm^2^.
2	Open lesion	Lesions where the skin and immediate subcutaneous layers are broken, including visible red tissue, dried or fresh blood, granulation tissue, lesions showing pus or lesions which appear moist due to fluids seeping from the skin.
3	Deep lesion	Lesions deep enough to show muscle, tendon, or bone.

**Table 3:** Item-wise monthly sale per household of recyclable waste in kilograms.

Recyclable Waste Sold per Month	Median (kg)	Range (kg)
Paper	1200	0–2400
Glass	450	0–14,400
Plastic	450	0–15,000
Plastic bottles	300	0–18,000
Bread	150	0–3000
Bone	90	0–4000
Hair	0	0–150
Wood	0	0–6000
Scrap metal	0	0–2000
Food residues	0	0–1800
Aluminium	0	0–450
Sold as such in bulk	0	0–36,000

**Table 4:** Number of donkeys suffering from superficial and open lesions. Deep lesions were not reported.

Lesion Location	Superficial or Healed Lesion		Open Lesion	
	Number of Donkeys	Percentage	Number of Donkeys	Percentage
Head/ear	8	3.9	28	13.7
Neck	0	0	11	5.4
Breast/shoulder	21	10.3	67	32.8
Fore leg	6	2.9	10	4.9
Knee	136	66.7	6	2.9
Wither/spine	12	5.9	67	32.8
Ribs/flank	9	8.8	25	6.4
Girth/belly	27	13.2	26	12.8
Hindquarter	11	5.4	64	31.4
Hind leg	5	2.4	20	9.8
Tail/tail base	10	4.9	3	1.5

## Supplementary Materials

The following are available online at https://www.mdpi.com/2076-2615/9/4/159/s1. Supplementary Material 1: Donkey owner questionnaire; Supplementary Material 2: Household questionnaire; Supplementary Material 3: Town committee questionnaire; Supplementary Material 4: Service provider questionnaire.
