# Supplementary material for: The Role and Welfare of Cart Donkeys Used in Waste Management in Karachi, Pakistan
Source: Animals (Basel). 2019 Apr 12;9(4):159. doi: 10.3390/ani9040159 (PMC6523980; doi:10.3390/ani9040159)
Supplement: Supplementary file 1 [file animals-09-00159-s001.zip › animals-465014-sup4/Supplementary Material 1.docx]

| Ref# | Interviewee name | Date | Time | Location# |
| --- | --- | --- | --- | --- |

**Supplementary Material 1.** Donkey owner questionnaire

| **1** | How many donkeys do you have? | | | | *1* | | *2* | | *3* | | | *4* | *5* | | | *other (please specify)* | | |
| --- | --- | --- | --- | --- | --- | --- | --- | --- | --- | --- | --- | --- | --- | --- | --- | --- | --- | --- |
| **2** | How many donkeys do you use to collect waste? | | | | *1* | | *2* | | *3* | | | *4* | *5* | | | *other (please specify)* | | |
| **3** | How many waste-collection carts do you have? | | | | *1* | | *2* | | *3* | | | *4* | *5* | | | *other (please specify)* | | |
| **Section 1** | | | | | | | | | | | | | | | | | | |
| **4** | What was the problem with your donkey the last time it was ill? |  | | | | | | | | | | | | | | | | |
| **5** | What did you do the last time that your donkey was ill? (select all that apply) | *Treated it myself* | | *Government vet* | | | | | | *Private vet* | | | | | | | *Government paravet* | |
|  |  | *Private paravet* | | *Unlicensed service provider (e.g. quack)* | | | | | | | | | | | | | *Nothing* | |
|  |  | *Other (specify)* | | | | | | | | | | | | | | | *NA* | |
| **6** | Which service providers are you aware of in this area? (select all that apply) | *Government vet* | | *Private vet* | | | | *Government paravet* | | | | | | | | | *Private paravet* | |
|  |  | *Unlicensed service provider (e.g. quack)* | | | | | | | | | *None* | | | *Other (specify)* | | | | |
| **7** | Why did you last contact each service provider you described in the previous question? | *Service provider (as above)* | | | *Reason (e.g. to treat a case, vaccination, deworming, never)* | | | | | | | | | | | | | |
|  |  |  | | |  | | | | | | | | | | | | | |
|  |  |  | | |  | | | | | | | | | | | | | |
|  |  |  | | |  | | | | | | | | | | | | | |
|  |  |  | | |  | | | | | | | | | | | | | |
| **8** | Are you satisfied with the animal healthcare services available for your donkey? | *Yes* | | | | | | | | | | | | | | | | |
|  |  | *No* | | | | | | | | | | | | | | | | |
| **9** | Why did you give this answer to question 8? |  | | | | | | | | | | | | | | | | |
| **10** | Why do you choose each service provider that you use (as mentioned in question 7)? (select all that apply)  (cross out any service providers that do not apply) |  | *Government*  *Vet* | | | *Private*  *Vet* | | | | *Government*  *Paravet* | | | | | *Private*  *paravet* | | *Quack* | *Other (specify)* |
|  |  | *Easily available* |  | | |  | | | |  | | | | |  | |  |  |
|  |  | *Cheap* |  | | |  | | | |  | | | | |  | |  |  |
|  |  | *Close by* |  | | |  | | | |  | | | | |  | |  |  |
|  |  | *Animal gets better following treatment* |  | | |  | | | |  | | | | |  | |  |  |
|  |  | *Other (specify)* |  | | |  | | | |  | | | | |  | |  |  |
|  |  | *I never use any service provider* | | | | | | | | | | | | | | | | |
| **11** | What are the reasons that stop you from using any of the service providers that are available (as mentioned in question 6)? (select all that apply) (cross out any service providers that do not apply) |  | *Government*  *Vet* | | | *Private*  *Vet* | | | | *Government*  *Paravet* | | | | | *Private*  *paravet* | | *Quack* | *Other (specify)* |
|  |  | *Not available* |  | | |  | | | |  | | | | |  | |  |  |
|  |  | *Too expensive* |  | | |  | | | |  | | | | |  | |  |  |
|  |  | *Too far away* |  | | |  | | | |  | | | | |  | |  |  |
|  |  | *Treatment won’t work* |  | | |  | | | |  | | | | |  | |  |  |
|  |  | *Other (specify)* |  | | |  | | | |  | | | | |  | |  |  |
|  |  | *No service providers are available* | | | | | | | | | | | | | | | | |

| **12** | | Have any of the donkeys that you use to collect waste died in the last two years? | | *Yes: How many?* | | | *1* | | | | | *2* | | | *3* | | | | | | *4* | *5* | | *Other (specify)* | | | | | |
| --- | --- | --- | --- | --- | --- | --- | --- | --- | --- | --- | --- | --- | --- | --- | --- | --- | --- | --- | --- | --- | --- | --- | --- | --- | --- | --- | --- | --- | --- |
|  |  |  |  | *No* | | | | | | | | | | | | | | | | | | | | | | | | | |
| **13** | | If the answer to 12 is yes, then what was the reason for each death? | | *1* | | | | |  | | | | | | | | | | | | | | | | | | | | |
|  |  |  |  | *2* | | | | |  | | | | | | | | | | | | | | | | | | | | |
|  |  |  |  | *3* | | | | |  | | | | | | | | | | | | | | | | | | | | |
|  |  |  |  | *4* | | | | |  | | | | | | | | | | | | | | | | | | | | |
|  |  |  |  | *5* | | | | |  | | | | | | | | | | | | | | | | | | | | |
|  |  |  |  | *Other (specify)* | | | | |  | | | | | | | | | | | | | | | | | | | | |
| **14** | If available, would you use a water trough located at the rubbish tip? | | | *Yes* | | | | | | | | | | | | | | | | | | | | | | | | | |
|  |  |  |  | *No* | | | | | | | | | | | | | | | | | | | | | | | | | |
| **15** | What worries you the most about looking after your donkey? What is the biggest constraint to having a fit and healthy donkey? (ask one in five respondents) | | |  | | | | | | | | | | | | | | | | | | | | | | | | | |
| **Section 2** | | | | | | | | | | | | | | | | | | | | | | | | | | | | | |
| **16** | Where do you collect waste from? | |  | | | | | | | *Area 1* | | | | | | *Area 2* | | | | | | | *Area 3* | | | | | | *Area 4* |
|  |  |  | *District* | | | | | | |  | | | | | |  | | | | | | |  | | | | | |  |
|  |  |  | *Town committee* | | | | | | |  | | | | | |  | | | | | | |  | | | | | |  |
| **17** | How many kilogrammes of non-recyclable waste do you dump daily on average? | |  | | | | | | | | | | | | | | | | | | | | | | | | | | |
| **18** | How many kilograms of recyclable waste do you collect daily on average? | |  | | | | | | | | | | | | | | | | | | | | | | | | | | |
| **19** | How much of each of the following categories of waste did you sell last time? | |  | | | | | | | | | | *Amount (kg)* | | | | | | | *Time taken to collect this amount of waste (days)* | | | | | | | | *Rate/kg* | |
|  |  |  | *Plastic* | | | | | | | | | |  | | | | | | |  | | | | | | | |  | |
|  |  |  | *Plastic bottles* | | | | | | | | | |  | | | | | | |  | | | | | | | |  | |
|  |  |  | *Paper* | | | | | | | | | |  | | | | | | |  | | | | | | | |  | |
|  |  |  | *Glass* | | | | | | | | | |  | | | | | | |  | | | | | | | |  | |
|  |  |  | *Scrap metal* | | | | | | | | | |  | | | | | | |  | | | | | | | |  | |
|  |  |  | *Aluminium* | | | | | | | | | |  | | | | | | |  | | | | | | | |  | |
|  |  |  | *Food residues* | | | | | | | | | |  | | | | | | |  | | | | | | | |  | |
|  |  |  | *Bread* | | | | | | | | | |  | | | | | | |  | | | | | | | |  | |
|  |  |  | *Bones* | | | | | | | | | |  | | | | | | |  | | | | | | | |  | |
|  |  |  | *Other (specify)* | | | | | | | | | |  | | | | | | |  | | | | | | | |  | |
|  |  |  | *Other (specify)* | | | | | | | | | |  | | | | | | |  | | | | | | | |  | |
| **20** | Where do you dump your non-recyclable waste? | |  | | | | | *Location 1* | | | | | | | | | | | | *Location 2* | | | | | *Location 3* | | | | |
|  |  |  | *Official* | | | | |  | | | | | | | | | | | |  | | | | |  | | | | |
|  |  |  | *Unofficial* | | | | |  | | | | | | | | | | | |  | | | | |  | | | | |
| **21** | Fill out this diagram to obtain the following information: (if distances are not known how long in minutes does it take?) | | | *Distance (km):*  *Time (mins):*  *Number of Trips:*  *Number of journeys (daily):*  *Distance (km):*  *Time (mins):*  *Number of Trips:*  *Number of journeys (daily):*  *Distance (km):*  *Time (mins):*  *Number of Trips:*  *Number of journeys (daily):* | | | | | | | | | | | | | | | | | | | | | | | | | |
| **A** | How far away from here is the place that you collect waste from in kilometres? | | |  |  |  |  |  |  |  |  |  |  |  |  |  |  |  |  |  |  |  |  |  |  |  |  |  |  |
| **B** | What is the distance between the place that you collect waste from and the rubbish tip in kilometres? | | |  |  |  |  |  |  |  |  |  |  |  |  |  |  |  |  |  |  |  |  |  |  |  |  |  |  |
| **C** | How far away from here is the rubbish tip in kilometres? | | |  |  |  |  |  |  |  |  |  |  |  |  |  |  |  |  |  |  |  |  |  |  |  |  |  |  |
| **D** | How many trips between these locations do you do daily? | | |  |  |  |  |  |  |  |  |  |  |  |  |  |  |  |  |  |  |  |  |  |  |  |  |  |  |
| **22** | What are the biggest challenges that you face in undertaking your waste collection job? (ask one in five respondents) | | |  | | | | | | | | | | | | | | | | | | | | | | | | | |
| **Section 3** | | | | | | | | | | | | | | | | | | | | | | | | | | | | | |
| **23** | How many people are in your household? | | |  | | | | | | | | | | | | | | | | | | | | | | | | | |
| **24** | Who from the family goes out to collect waste each day? (categories describe relationship to head of household) | | |  | | | | | | | *Number* | | | | | | | *Age<18 years* | | | | | | | | | *Age<14 years* | | |
|  |  |  |  | *Household head* | | | | | | |  | | | | | | |  | | | | | | | | |  | | |
|  |  |  |  | *Father* | | | | | | |  | | | | | | |  | | | | | | | | |  | | |
|  |  |  |  | *Son/Nephew* | | | | | | |  | | | | | | |  | | | | | | | | |  | | |
|  |  |  |  | *Brother* | | | | | | |  | | | | | | |  | | | | | | | | |  | | |
|  |  |  |  | *Daughter* | | | | | | |  | | | | | | |  | | | | | | | | |  | | |
|  |  |  |  | *Sister* | | | | | | |  | | | | | | |  | | | | | | | | |  | | |
|  |  |  |  | *Hired labourer* | | | | | | |  | | | | | | |  | | | | | | | | |  | | |
|  |  |  |  | *Other(specify)* | | | | | | |  | | | | | | | | | | | | | | | | | | |
| **25** | Who is involved in grading and selling the recyclable waste? (categories describe relationship to head of household) | | |  | | | | | | | *Number* | | | | | |  | | | | | | | | | | | | |
|  |  |  |  | *Household head* | | | | | | |  | | | | | |  | | | | | | | | | | | | |
|  |  |  |  | *Father* | | | | | | |  | | | | | |  | | | | | | | | | | | | |
|  |  |  |  | *Mother* | | | | | | |  | | | | | |  | | | | | | | | | | | | |
|  |  |  |  | *Wife* | | | | | | |  | | | | | |  | | | | | | | | | | | | |
|  |  |  |  | *Son/nephew* | | | | | | |  | | | | | |  | | | | | | | | | | | | |
|  |  |  |  | *Daughter* | | | | | | |  | | | | | |  | | | | | | | | | | | | |
|  |  |  |  | *Brother* | | | | | | |  | | | | | |  | | | | | | | | | | | | |
|  |  |  |  | *Sister* | | | | | | |  | | | | | |  | | | | | | | | | | | | |
|  |  |  |  | *Hired labourer* | | | | | | |  | | | | | |  | | | | | | | | | | | | |
|  |  |  |  | *Other (specify)* | | | | | | |  | | | | | |  | | | | | | | | | | | | |
|  |  |  |  | *Other(specify)* | | | | | | |  | | | | | |  | | | | | | | | | | | | |
| **26** | How many households in your area(s)? | | | Area 1: ____________ Area 2:_____________ Area 3: _________________ | | | | | | | | | | | | | | | | | | | | | | | | | |
| **27** | How much household pay for waste management monthly? | | | Area 1: ____________ Area 2:_____________ Area 3: _________________ | | | | | | | | | | | | | | | | | | | | | | | | | |
| **28** | How much money do you get from the town committee each month? | | |  | | | | | | | | | | | | | | | | | | | | | | | | | |
| **29** | How much did you pay for this donkey? | | |  | | | | | | | | | | | | | | | | | | | | | | | | | |
| **30** | How much did you pay for this cart and harness? | | |  | | | | | | | | | | | | | | | | | | | | | | | | | |
| **31** | How much does it cost to feed your donkey each day? | | |  | | | | | | | | | | | | | | | | | | | | | | | | | |
| **32** | How much do you usually spend monthly on medicines and healthcare? | | |  | | | | | | | | | | | | | | | | | | | | | | | | | |
| **33** | Is there anything thing else that you have to buy for your donkey? How much does it cost? | | | *Item* | | | | | | | | | | *Cost* | | | | | | | | | | | | | | | |
|  |  |  |  | *Shoeing* | | | | | | | | | |  | | | | | | | | | | | | | | | |
|  |  |  |  | *Clipping* | | | | | | | | | |  | | | | | | | | | | | | | | | |
|  |  |  |  | *Cart maintenance* | | | | | | | | | |  | | | | | | | | | | | | | | | |
|  |  |  |  | *Harness maintenance* | | | | | | | | | |  | | | | | | | | | | | | | | | |
|  |  |  |  | *Any other specify* | | | | | | | | | |  | | | | | | | | | | | | | | | |
| **34** | Does your household have any other sources of income? What are they? | | | *No* | | *Yes (specify)* | | | | | | | | | | | | | | | | | | | | | | | |
| **35** | What proportion of your household’s income comes from waste management? | | | *One quarter* | | | | | *One half* | | | | | | | | | | *Three quarters* | | | | | | | *All* | | | |
| **36** | Do you know of any other NGOs working in this location? | | | *No* | *Yes (specify)* | | | | | | | | | | | | | | | | | | | | | | | | |
| **37** | What would happen to you and your family of your donkey dies? | | |  | | | | | | | | | | | | | | | | | | | | | | | | | |
