# Supplementary material for: The Role and Welfare of Cart Donkeys Used in Waste Management in Karachi, Pakistan
Source: Animals (Basel). 2019 Apr 12;9(4):159. doi: 10.3390/ani9040159 (PMC6523980; doi:10.3390/ani9040159)
Supplement: Supplementary file 1 [file animals-09-00159-s001.zip › animals-465014-sup4/Supplementary Material 2.docx]

| Ref# | Interviewee name | Date | Address |
| --- | --- | --- | --- |

Supplementary Material 2. **Household questionnaire**

| **1** | What sort of property is this? | *Residential* | | | | | *Shop* | | | *Hospital* | | |
| --- | --- | --- | --- | --- | --- | --- | --- | --- | --- | --- | --- | --- |
|  |  | *Factory* | | | | | *Market* | | | *Other (specify)* | | |
| **2** | How frequently do waste-collecting donkey carts visit you? | *Daily* | | | | *Weekly* | | | *Monthly* | | | *Other (specify)* |
| **3** | How much do you pay for garbage disposal per month? | *Donkey carts* | | | | | | | | | | |
|  |  | *Other (specify)* | | | | | | | | | | |
| **4** | Who do you pay for garbage disposal? | *Cart Owner* | | | | | *Town Committee* | | | | *Other (specify)* | |
| **5** | What happens if the donkey cart does not visit your house? |  | | | | | | | | | | |
| **6** | Do you ever have to dispose of your waste yourself? | *No* | *If yes:* | | *Why?* | | | | | | | |
|  |  |  |  | | *Where do you take it?* | | | | | | | |
|  |  |  |  | | *Rate how inconvenientthis is (rate 1 to 10, where 10 is the most inconvenient)* | | | | | | | |
| **7** | What would you do if the donkey carts stopped working in your area? |  | | | | | | | | | | |
| **8** | Does any other method of waste collection occur for your property? |  | | | | | | *Reason / NA if does not occur* | | | | |
|  |  | *Karachi Municipality Corporation Truck* | | | | | |  | | | | |
|  |  | *Suzuki pick-up* | | | | | |  | | | | |
|  |  | *Auto-rickshaw* | | | | | |  | | | | |
|  |  | *Bicycle* | | | | | |  | | | | |
|  |  | *Other (specify* | | | | | |  | | | | |
| **9** | Which sort of waste collector would you prefer for your property? |  | | | | | | *Reason* | | | | |
|  |  | *Donkey cart* | | | | | |  | | | | |
|  |  | *Karachi Municipality Corporation Truck* | | | | | |  |  |  |  |  |
|  |  | *Suzuki pick-up* | | | | | |  |  |  |  |  |
|  |  | *Auto-rickshaw* | | | | | |  |  |  |  |  |
|  |  | *Bicycle* | | | | | |  |  |  |  |  |
|  |  | *Other (specify* | | | | | |  |  |  |  |  |
| **10** | What would your ideal garbage disposal system be? Which department/ organisation should be responsible for implementing this? |  | | | | | | | | | | |
|  |  | *Responsibility:* | | | | | | | | | | |
| **11** | Do you think that donkeys should be used in waste management? Why? | *No* | | *Yes (specify)* | | | | | | | | |
