# Supplementary material for: The Role and Welfare of Cart Donkeys Used in Waste Management in Karachi, Pakistan
Source: Animals (Basel). 2019 Apr 12;9(4):159. doi: 10.3390/ani9040159 (PMC6523980; doi:10.3390/ani9040159)
Supplement: Supplementary file 1 [file animals-09-00159-s001.zip › animals-465014-sup4/Supplementary Material 3.docx]

**Supplementary Material 3.** Town committee questionnaire

| Ref# | Town committee name | Date | Interviewee name and position in committee |
| --- | --- | --- | --- |

How is waste management administered in this area? (amount of waste daily, different stakeholders involved, relationship between public and private enterprises, public opinion)

What is the town committee’s role in waste management? (processes, responsibilities, collection, type of relationship with stakeholders involved)

Which waste collectors does this town committee hire (list all that apply).

Why does the town committee hire these waste collectors? (advantages and disadvantages of each group)

What is the town committee’s relationship with the waste-collecting cart donkey owners (process, contract, regulating the process, backup plan)

Which government department is responsible for waste management?

What is the town committee’s relationship with KMC, EPA and other organisations (for example at provincial level)? (contracts, responsibilities, political, processes etc.)

Which waste management sites are under the town committee’s control (number of dumping sites, location, what aspects are controlled by the committee)

Are you aware of any other waste management suites within the town committee’s area? (regulated or unregulated, opinion, control options)

Is there any policy about the collection and carriage for waste?

What challenges does the town committee face in waste management? (how would they like to overcome them)

What role do you think that donkey carts have in waste management in Karachi City as a whole?

Do you think this role will change over the next five years? (How? Why?)
