# Supplementary material for: The Role and Welfare of Cart Donkeys Used in Waste Management in Karachi, Pakistan
Source: Animals (Basel). 2019 Apr 12;9(4):159. doi: 10.3390/ani9040159 (PMC6523980; doi:10.3390/ani9040159)
Supplement: Supplementary file 1 [file animals-09-00159-s001.zip › animals-465014-sup4/Supplementary Material 4.docx]

**Supplementary Material 4.** Service provider questionnaire

| Ref# | Interview location | Date | Interviewee name |
| --- | --- | --- | --- |

What qualifications do you have? (list all relevant training, training establishment attended, current job title)

Where do you practice veterinary medicine? (clinic/shop location, distance and method travelled to cases, other regular locations e.g. tonga stands)

How many cases do you see each month (include type of case e.g. preventative / reproduction / clinical case and animal species)

What are the most common cases that you see in working equids?

How much do you charge to see a donkey? (minimum and maximum amount, brief overview of pricing structure)

Do you provide services to the equine owners in Jamali Goth? (which services, for how long, how often)

What are the most common cases that you see in the donkeys from Jamali Goth? (type of case, frequency of case)

What challenges do you face when treating the donkeys from Jamali Goth?
